# Deep Learning-Based Positioning of Visually Impaired People in Indoor Environments

**DOI:** 10.3390/s20216238

**Published:** 2020-10-31

**Authors:** Payal Mahida, Seyed Shahrestani, Hon Cheung

**Affiliations:** School of Computer, Data and Mathematical Sciences, Western Sydney University, Second Ave, Kingswood 2747, Australia; S.Shahrestani@westernsydney.edu.au (S.S.); H.Cheung@westernsydney.edu.au (H.C.)

**Keywords:** indoor, positioning, visually impaired, deep learning, multi-layered perceptron, inertial sensor, smartphone

## Abstract

Wayfinding and navigation can present substantial challenges to visually impaired (VI) people. Some of the significant aspects of these challenges arise from the difficulty of knowing the location of a moving person with enough accuracy. Positioning and localization in indoor environments require unique solutions. Furthermore, positioning is one of the critical aspects of any navigation system that can assist a VI person with their independent movement. The other essential features of a typical indoor navigation system include pathfinding, obstacle avoidance, and capabilities for user interaction. This work focuses on the positioning of a VI person with enough precision for their use in indoor navigation. We aim to achieve this by utilizing only the capabilities of a typical smartphone. More specifically, our proposed approach is based on the use of the accelerometer, gyroscope, and magnetometer of a smartphone. We consider the indoor environment to be divided into microcells, with the vertex of each microcell being assigned two-dimensional local coordinates. A regression-based analysis is used to train a multilayer perceptron neural network to map the inertial sensor measurements to the coordinates of the vertex of the microcell corresponding to the position of the smartphone. In order to test our proposed solution, we used IPIN2016, a publicly-available multivariate dataset that divides the indoor environment into cells tagged with the inertial sensor data of a smartphone, in order to generate the training and validating sets. Our experiments show that our proposed approach can achieve a remarkable prediction accuracy of more than 94%, with a 0.65 m positioning error.

## 1. Introduction

Accessible location-based information for navigating in a complex indoor environment is a need of every individual [1]. Navigation in complex infrastructures, such as shopping malls, airports, and hospitals, is aided by the proliferation of visual maps, digital maps, and kiosks. However, visually impaired (VI people) can find it hard to use such aids effectively. Globally, 285 million people are estimated to be visually impaired. Amongst them, 39 million are legally blind. Mobility and being able to move around independently can pose significant challenges for a VI person [2].

When travelling in a new environment or public buildings, VI people may require directional assistance or some form of navigation aid. A robust outdoor navigation solution is provided by the Global Positioning System (GPS). However, the use of GPS in an indoor environment is not always possible, as the satellite signals that they rely on cannot penetrate most walls [3]. Special technologies—such as raised line maps, i.e., tactile maps and signage information in Braille—can be of assistance to a VI person in a complex environment [4]. However, not all VI individuals can read and understand the tactile maps [5]. Apart from that, the tactile maps have limitations, including static information about the changing surroundings, and the difficulty of a VI person to position themselves [6]. A white cane is a luminous physical aid that allows a VI person to scan their surrounding for obstacles. However, it fails to identify the location of the individual. The development of innovative modern technologies, such as the Internet of Things and artificial intelligence, has opened up possibilities for providing an interactive system to assist a VI person to independently navigate in an indoor environment.

The lack of a robust technology hinders the navigation of a VI person due to several issues regarding the layout complexity, accessibility, connectivity, and temporal changes of the environment [7]. Technologies need to ease the processes of VI people’s navigation by solving challenging issues like the provision of suitable indoor positioning, the tracking of the moving users, obstacle avoidance, and pathfinding [8]. Currently, a variety of wireless technologies are available for indoor positioning and navigation, relying on ZigBee, Radio Frequency Identification (RFID), Beacon, Bluetooth, ultra-wideband (UWB) radio, magnetic fields, and pedestrian dead reckoning (PDR) [8,9].

In our previous works, we have reported on the provision of solutions for the movements of VI people in a smart environment using interconnected IoT devices [10]. A robust framework is the utilization of Bluetooth low energy beacon sensors in the building in order to help a VI person navigate indoors. A developed algorithm, DynaPATH, generates VI-friendly safe routes to a destination, considering significant vision constraints, such as walking along the walls, and the creation of a straight path with minimal turns. Unlike solutions that choose the shorter path [11], DynaPATH proposes a safe path considering the limitations of the VI people [12]. However, a VI person may find difficulty in positioning him/herself in an open space—such as a big hallway—due to the unavailability of external physical devices. Due to the possible loss of external signals, the system needs to maintain the position of the VI people when other external devices are out of range. This paper addresses this positioning issue and investigates the use of inertial sensors to provide a complementary solution that can be integrated into our work. The contribution of this article is to provide a self-directed, accurate, and audio-aided standalone positioning system considering the constraints of a VI person. The main idea behind the work is to demonstrate the minimum infrastructure usage that can help VI people to overcome the challenge of positioning themselves independently between the landmarks.

**Figure 1:**
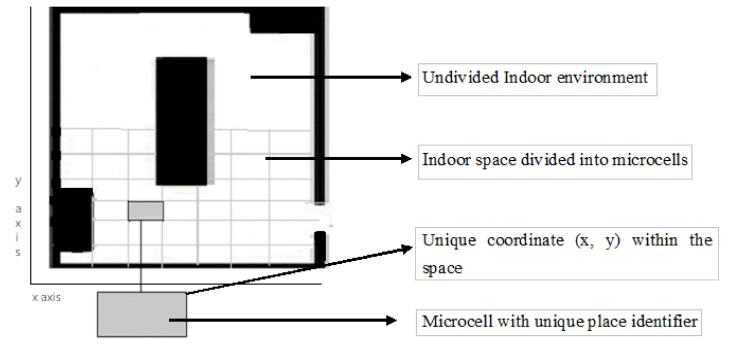
Grid distribution of an indoor environment.

This paper proposes a deep learning approach to the positioning of a VI user in an indoor environment with a smartphone as inertial guidance. Each room is given a room identifier. The indoor area is divided into microcells, each of which is assigned with a unique region/place identifier that acts as a recognition layer. The vertex of each microcell has 2D (*x*, *y*) local coordinates. Figure 1 shows the representation of a sample floor plan that has undivided and divided areas. The solid black lines represent the walls of the indoor environment, and the obstacles in the rooms are represented as solid filled rectangles. The indoor space is divided into grids of cells, as depicted in the lower part of the Figure. The shaded grey rectangle is a unique microcell with 4 vertices. The local coordinates are allocated manually and are stored for each vertex in the building, resembling the latitude and longitude used in a GPS.

In this paper, we propose to map the inertial sensor measurements of a smartphone into position coordinates using the regression-based training of a deep neural network (DNN). We report the results of various experiments in order to check the suitability of our proposed approach. Our experiments used a publicly available dataset that contains records that resemble the walking and movement data of a VI person, e.g., walking straight along the walls. The novelty of our reported approach stems from the use of regression-based multilayer perceptron (MLP) neural network training to accurately find the position of the VI person in a building.

**Figure 2:**
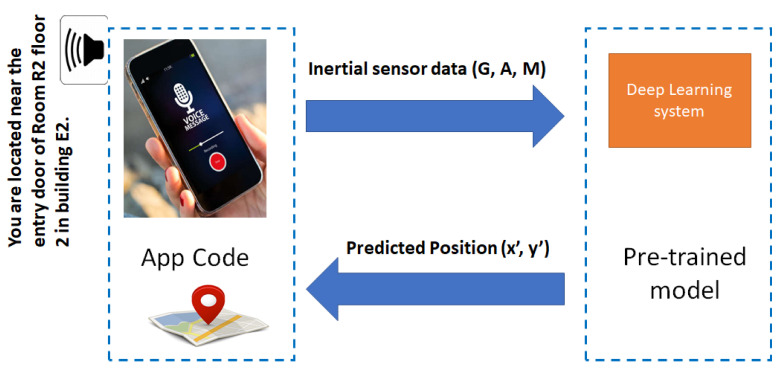
Interaction between the smartphone app and the pre-trained model.

After the validating experiments, we developed an application for the indoor positioning of a VI person. Figure 2 shows the interactions between the application residing in a smartphone and the pre-trained model established by a deep neural network. The inertial sensors of a smartphone provide the inputs to the deep neural network. The MLP will then use these measurements to estimate the corresponding position. The app informs the user of their relative position through an audio interface.

Our contributions in the article, summarized below, aim to provide a robust independent inertial guidance tool to position a VI person in an indoor environment:

- The work proposes an audio assistant app, to be developed and deployed on a smartphone, that helps VI people move independently in a complex building.
- To the best of the author’s knowledge, this work is the first to propose and recommend regression-based neural network training for the estimation of the position of a VI person moving in an indoor environment with a smartphone.
- We experimented with a deep neural network model to predict the position of an indoor user as a complementary system to our existing navigation framework using external sensors [10,12].

The remainder of this paper is organized as follows. Section 2 discusses the related research work that sought to solve the indoor positioning problem. The multivariate IPIN2016 dataset, with its usability to evaluate the 2D position of an indoor user, is introduced in Section 3. Furthermore, in Section 4, different versions of the regression-based deep neural network trainings are presented for experimentation. It discusses the deep network structure with the hyperparameters used in the experiments. Section 5 discusses the experimental platform and selection of deep network architecture with suitable hyperparameters, considering the prediction accuracy and the localization error. Finally, conclusions are drawn in Section 6, which also presents the limitations.

## 2. Background and Motivations for This Work

For a VI person, the localization error needs to be within a few centimetres in order to locate a user in the right room within a building. The system should also be able to estimate and update the location of the moving user quickly. The literature review of this work focuses on prediction accuracy, positioning error, and the usage of technologies that can use minimal resources.

The existing indoor navigation and positioning technologies for VI people can be categorized as being vison based, non-vision based, and based on IoT devices [8]. However, their popularity differs for inaccuracies due to indoor disturbances, availability, energy consumption, the cost of installation, and being high maintenance [13]. Considering these challenges, the performance of indoor location-based services highly depends on the appropriate choice of technology and approaches. Vison-based positioning technologies require the receivers and the moving object or person to be in the line of sight (LOS) to estimate the position measurements [14]. This category involves a vision-based camera and infrared ultrasonic systems [15]. Guerrero [16] suggested a micro navigation system using an infrared camera, Wiimotes, and an augmented white cane to detect the user’s position and movement. This system requires massive resources, and the computation operation needed to map and position the user is too great.

Non-vision-based positioning technology includes narrow and wideband wireless radio frequency and magnetic field-based technologies [14]. Indoor positioning has been attempted using WiFi (Wireless Fidelity), infrared, RFID, ultrasound, Bluetooth, and a combination of the technologies [17,18]. A Radial based network including Infrared and RFID has acceptable localization errors. However, it suffers from high costs, as it requires additional hardware and needs offensive calibration processes [15]. Ultrasound waves are used to estimate and track the position of a user in ultrasound-based systems. However, the blockage of the line of sight might result in incorrect measurements [19]. SUGAR [20] uses multiple UWB tags that achieve suitable localization errors for VI people; up to 38 cm. However, installing the UWB system is expensive, and the positioning is purely based on a UWB tag. Nakajima has proposed the use of visible light communication (VLC) and geomagnetic sensor to position and localize the user in an indoor environment [21]. The system provides localization errors up to 1 to 2 m, which are not sufficient for VI people.

Several attempts have been made to develop indoor navigation systems; however, not many of them are successfully deployed. NavCog is a smartphone-based turn-by-turn navigation system for blind users using a network of Bluetooth low energy (BLE) beacons, which uses a K-nearest neighbour (KNN) algorithm approach [22]. The system achieves precise localization information. However, the solution demands a rerouted path to the destination due to missed turns. LowViz [23] is the latest mobile application to assist the visually impaired in indoor navigation. The system uses a wide range of technologies—including sensors, WiFi and Bluetooth low-energy beacons—to guarantee a low localization error. However, context-aware real-time pathfinding is yet to be included in the system. The app may fail when the signals from external devices fail. A variety of newly-developed technologies are being generated and tested. Still, the designs suffer from limitations in their localization error, hardware cost, availability, and lack of additivity.

Recently, there has been considerable new interest in indoor localization techniques, driven by the proliferation of smartphones and other mobile devices. Traditional approaches, such as WiFi-based fingerprinting or distance-based methods, have low prediction accuracy due to shallow learning [24]. In order to handle the shallow learning problem, the deep neural network (DNN) is implemented for the self-extraction of appropriate low and high-level features of given raw data [25,26,27]. DNN approaches have shown good performance against signal fluctuations, noise effects, and time-consuming manual tuning [28]. The deep networks dynamically learn from the environment by mapping noisy and complex input data to the corresponding output [29]. To the best of the authors’ knowledge, not enough work has been done to provide deep learning-based positioning for VI people. Due to limitations in the positioning system for VI people, we have reviewed indirectly applicable positioning using WiFi, inertial sensors, and Channel State Information (CSI).

A novel indoor classification approach [24] was proposed with WiFi fingerprints to predict the correct floor and locations using a deep neural network. The work in [30] used heterogeneous network data, including WiFi and cellular networks with recurrent neural network algorithms, with a high average error of 9.19 m. The positioning error is approximately 9 m, which is not suitable for a VI person. A recurrent neural network (RNN) based indoor positioning solution [31] was applied to RSS data, exploiting the sequential correlation of RSS data. The work achieved an average localization error of 0.75 m, with 80% of the errors being below 1 m. The integration of Linear discriminate analysis (LDA) and MLP based on RSS was proposed in [28]. The approach has a 99.15% prediction accuracy, with a 0.98 m positioning error. RSS-based approaches have high variability at a fixed position in each time. Furthermore, RSS-based localization systems have coarse information due to multipath channels from different antennas. RSS-based approaches usually have 1–3 m of localization error, which it is difficult to further improve [32].

A localization technique based on CSI fingerprints collected using a single access point was proposed in [33]. It used a principal component analysis (PCA) feature extraction technique with different positioning errors in different rooms, varying between 0.6 m and 1.08 m. In [26], the authors compared the results of positioning using MLP and a convolutional network with RSS and CSI data. The RSS data could achieve an average of a 0.92 m localization error, with the highest error as 9 m. The results with the CSI data achieved a 0.92 m positioning error, with a maximal localization of 1.92 m. Besides WiFi network information, the magnetic field signals captured from the magnetometer are similar to the earth’s non-constant magnetic field [34]. Each building has its unique magnetic field, with some local anomalies. Thus, the static magnetic field can be utilized in indoor localization and navigation systems [34,35,36]. An RNN deep neural network [36] approach applied to magnetic signals indoors achieved a positioning localization error of 1.062 m, compared to an average error of 3.14 m with BLE fingerprinting results.

Despite extensive research, the algorithms and technologies mentioned above are still facing issues related to accuracies, infrastructure, and computational complexity. Most of the indoor solutions focus on the use of additional high-computing devices, including beacons and RFIDs. The decrease of the cost and size of the sensors and spurring technologies have resulted in smartphones as a useful and popular IoT device. Modern smartphones have several such sensors, including accelerometers, gyroscopes, magnetometers, GPS, gravity sensors, barometers, and ambient light sensors [37]. Considering the need of a VI person for indoor navigation, we have experimented with the use of the smartphone as navigation assistance in an indoor space.

From the related works studied, most of the positioning systems have focused on solving the underlying issue as a classification problem using WiFi signals by providing room-specific information. We aim to mitigate the infrastructure dependency to position a VI person by proposing the use of a commonly-carried device: a smartphone.

## 3. Characteristics of the Used Dataset

The use of an appropriate dataset for the training and testing of the model is an essential step in a deep neural network. Despite many works trying to solve the indoor localization issue, there is a lack of public datasets with inertial sensor data for a controlled environment. With the limited number of datasets, we have used a multivariate IPIN2016 dataset [38] in our work to test the proposed approach. Though the pedestrian collecting the inertial sensor data is not visually impaired, the movement of the user has similar steps, including walking along the wall and walking at the same pace.

Like our design, the dataset splits the indoor environment into cells mapped with the inertial sensor data of a smartphone. This Section discusses the dataset and its usability for a controlled indoor environment for VI people. The dataset has different types of movement fingerprints, including magnetic readings from smartphone/smartwatches in the divided spaces. Magnetic readings are data captured by the magnetometer, accelerometer, and gyroscope of a smartphone/smartwatch. The multivariate IPIN2016 dataset has captured the records of the moving user in 325 different places [38]. This dataset includes 36,795 continuous samples over two scenarios of one hour at 10 Hz, which resulted in 6500 discrete samples in 325 places.

**Figure 3:**
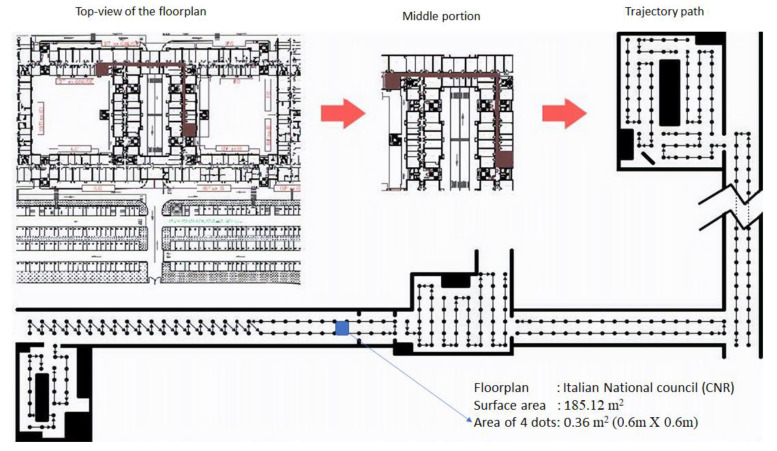
Indoor Floorplan with the top view and trajectory path from iPIN2016 [38].

The dataset was created on the first floor of the Institute of Information Science and Technologies (ISTL), inside the Italian National Council (CNR) building. The dataset covered movements on a surface measuring 185.12 m^2^. Figure 3 depicts the overall map of the building with the top view and trajectory path [38]. The top left corner of the Figure is the top view of the floorplan. The middle portion of the Figure is the highlighted corridor of the given floor.

Furthermore, the trajectory path followed by the users is shown in the bottom part with dots. Each dot in the map corresponds to a detection point, and each dot is 0.6 m from another. The dots represent the different locations at which two users acquired inertial sensor data on their smart devices. As such, the combination of each four dots occupies an area of 0.6 m × 0.6 m. Due to the fixed size of the microcell in the given dataset, our experiments use the same grid size. However, there is a further scope to observe the effects of different grid sizes on the results.

The dataset consists of two scenarios with a combination of zigzag and straight path trajectories performed by two different users holding a smartphone, in order to cover the entire target area. The walking speed of each user was 0.6 m/s on average. Each sample was collected roughly every 100 ms, and the collection time was short. The dataset is a unique combination of both WiFi signals and the inertial sensor data of both a smartphone and smartwatch. This study does not consider data from the WiFi access points and the smartwatch data. During the acquisition, the smartphone was kept at the chest level, with the screen facing up. Every time the user was at a specific location, the device recorded the following data at each dot location.

The recorded data includes the following readings at each dot, represented as a PlaceID with their local coordinates (*x*, *y*) at a given timestamp:

- *X*, *Y*, and *Z*-axis values of the accelerometer sensor;
- *X*, *Y*, and *Z*-axis values of the magnetometer;
- *X*, *Y*, and *Z*-axis values of the gyroscope;
- Roll, pitch, and azimuth values of the inertial sensor.

**Figure 4:**
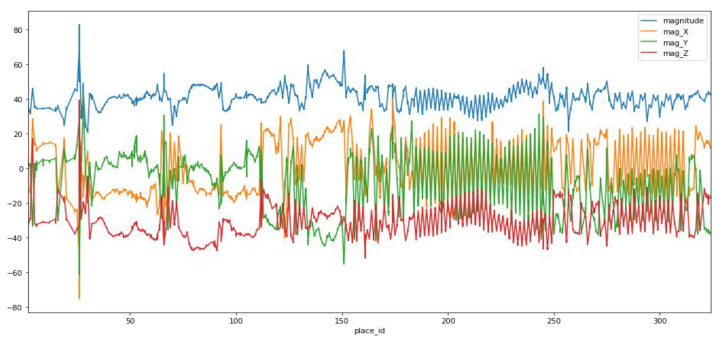
Graphical representation of the *x*, *y*, and *z* coordinates and magnitude of the magnetometer readings.

Our work focuses on the magnetic field signals of the accelerometer and gyroscope of the smartphone. Figure 4 represents the graphical representation of values from the *x*, *y* and *z*-axis of a magnetometer. The normalized magnitude M_mag_ of the magnetometer is calculated by Equation (1).

(1) $$M_{\mathrm{mag}}=\sqrt{M_{x}{}^{2}+M_{y}{}^{2}+M_{z}{}^{2}}$$

where M_mag_ is the normalized magnitude of the magnetometer. *M_i_* is the value of the *i*th axis of the 3-axis accelerometer.

**Figure 5:**
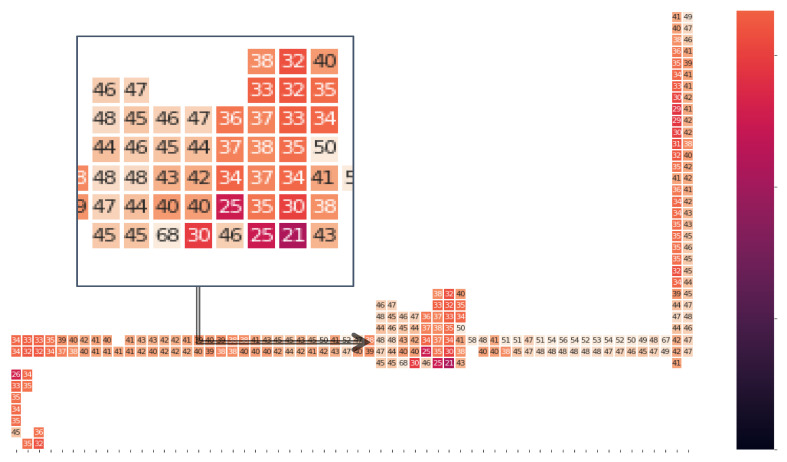
Heatmap of the magnitude of the magnetic field in each place in the building.

Figure 5 shows the magnetic field heatmap in each location on the trajectory of the corridor, followed by user 1. The number represented in each cell of the grid on the heatmap is the normalized magnitude, as evaluated in Equation (1). The value of the magnitude varies from 21 to 68 for the given dataset. The indoor magnetic field may be distorted over time locally because of the steel-reinforced concrete in the structures. However, the study in [39] reveals that the magnetic field’s distortion pattern remains static.

## 4. Deep Learning-Based Positioning 

This Section discusses the detailed deep neural network model proposed to predict and evaluate the position of a moving user in a controlled environment. The MLP is characterized as fully connected layers, where each perceptron relates to every other perceptron. The MLP model is a class of feedforward artificial network that defines a mapping function, as shown in Equation (2) [29]:

(2) $$y=\psi \left(\overset{n}{\underset{i=1}{\sum }}\omega_{i}x_{i}+b\right)=\psi \left(w^{T}x+b\right)$$

where *y* is the target, *w* denotes the vector of the weights, *x* is the vector of the inputs, *b* is the bias, and ψ is a non-linear activation function.

In this work, we propose to use a regression-based training algorithm to generate the MLP weights, mapping the inertial sensor data of a smartphone into the coordinates of the phone.

**Figure 6:**
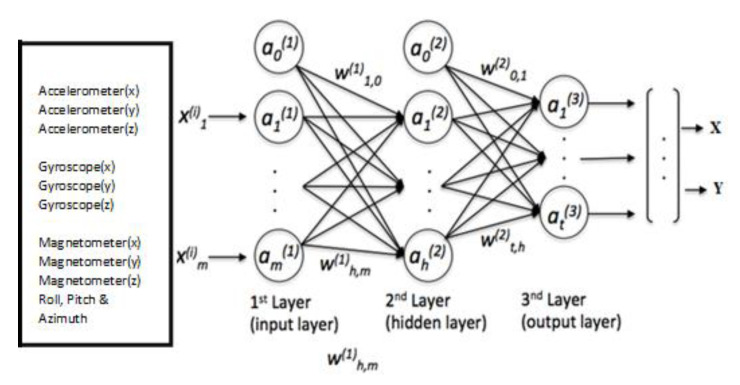
The MLPv1 network structure.

In this case, the inputs of the MLP correspond to the 3-axis inertial sensor measurements. The output layer delivers the coordinates of a point in two-dimensional space: *x* and *y*. Figure 6 represents the MLP-based DNN with three hidden layers consisting of 128, 64 and 128, neurons used in this work.

In order to mitigate the effect of unstable gradients with the given neural network, an additional batch normalization layer was introduced to perform an optimization on the input layers. The batch normalization layer works by performing a series of operations on the incoming input data [29]. For equal distribution amongst the input of the hidden layers and faster convergence, we adopted the batch normalization layer between the hidden layers. The weights of the hidden layer are updated by a reduction in the loss function L, as expressed in Equation (3) using the back-propagation algorithm.

(3) $$L=\frac{1}{m}\overset{m}{\underset{i=1}{\sum }}{\left(yi-f\left(xi\right)\right)}^{2}$$

where *m* represents the number of samples of input features, and *yi* represents the actual coordinates of the *i*th sample. *f*(*xi*) is a function to predict the position from the *i*th sample of the input features.

**Figure 7:**
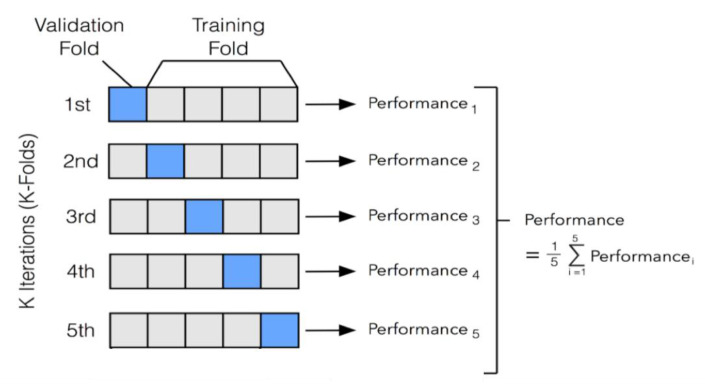
k-Fold Cross-validation technique.

The data is split into two subsets to train the MLP and to validate the learning. The testing of the performance involves the use of an independent dataset that was not used for the training of the model. The size of the dataset in our simulation is comparatively small and intricate. Therefore, we used the K-Fold Cross-validation technique. The K-fold method is a resampling procedure used to evaluate a deep learning model based on a limited number of data samples [40]. It is popular because it is a less biased or a less optimistic estimate of the model than a simple train/test split. This technique involves the random division of a dataset into K groups, or folds, of approximately equal size. The first blue fold is treated as validation data, and the model is trained on the remaining K-1 training data, as depicted in the first of the K iterations in Figure 7. A validation fold is used to monitor the performance during training and is not used in training the model. In the second iteration, the second fold is used as validation data, while the rest are used in the training process, and so on.

The distribution of the training and validation data in the experiments is with k = 5 in the K-fold cross-validation technique. The dataset is equally distributed in five parts, including the first 7359 records as the validation data, and the remaining 29,436 as training data in the first iteration. Five iterations are performed over the total samples each time, in which 7359 data samples are treated as validation, and the remaining are treated as training data.

The training data is used in each iteration with fixed hyperparameters. Hyperparameters are the higher-level properties of the data model that improves the performance of the model and conveys the capacity of the model to learn the complexity of the data [41]. In order to improve the performance of the model, we involved hyperparameters, including several layers, epochs, a mini-batch size, an activation function, a dropout, regularization, and optimizers [42]. The experimental model was implemented in python, with Keras and TensorFlow libraries with different settings and hyperparameters, as listed in Table 1. 

The experiments were performed with different hyperparameters settings making a different version of the MLP model. Furthermore, the training data and labels are tuned to select the final model. The training data is tuned with the best hyperparameters and learning algorithms. The next Section discusses the experimental results of the different settings with the best-suited hyperparameters.

## 5. Setup of the Experiments and Analysis of the Results

The experimental platform used to the test the performance of the proposed neural network is presented in Section 5.1, and the performance metrics and evaluation results are discussed in Section 5.2.

### 5.1. Experimental Platform

**Figure 8:**
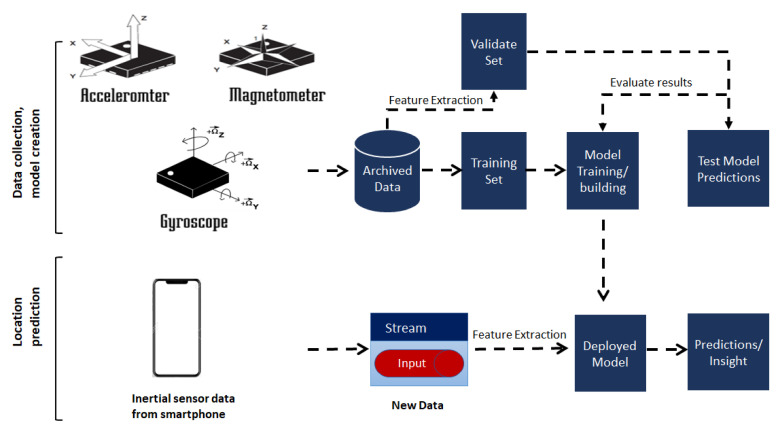
Experimental platform for the proposed model.

Figure 8 represents the experimental platform in the context of the estimation of the position of the user using a deep MLP algorithm.

The experimental platform expects the sequence of inertial sensor values, including the accelerometer, gyroscope, and magnetometer from the dataset. The collected values are archived and passed as the training set to build the model. The model expects live stream data from a smartphone. Furthermore, the features are extracted and passed to the model for the prediction of the position.

In this work, a sequence of inertial sensor sample values from a dataset—including those from accelerometer, gyroscope, and magnetometer for a moving user—are collected and fed as an input to the neural network as training data. We train the neural network as a regression problem in order to learn the 2-dimensional location of the user based on the input information. After the training, a model is established, and it can be used to estimate a user’s location based on real-time sensor data. In order to test the performance of the model, a test set based on the K-fold technique is used to evaluate the prediction accuracy of the proposed model.

### 5.2. Performance Metrics and Evaluation

The deep learning model is implemented using python, with libraries such as TensorFlow and Scikit-learn. The performance is measured using the mean squared distance (MAE), root mean squared error (RMSE), and mean squared error (MSE) between the ground truth and the predicted location. The model’s evaluation process is the assessment of the localization error and the prediction accuracy of a model on the multivariate IPIN2016 dataset, as described in Section 3. The MAE is the mean of the absolute value of the errors, as shown in Equation (4).

(4) $$MAE=\frac{1}{n}\overset{n}{\underset{i=1}{\sum }}\left|PS_{i}-SA_{i}\right|$$

The mean squared error (MSE) measures the average of the squares of the errors. It is the average squared difference between the actual value and the estimated value, as shown in Equation (5).

(5) $$MSE=\frac{1}{n}\overset{\Pi }{\underset{i=1}{\sum }}{\left(PS_{i}-SA_{i}\right)}^{2}$$

The RMSE is a measure of the average deviation of the predicted values from the actual values. It is used to measure the difference between the values predicted by a model and the values observed from the modelled environment. The average localization error of the calculated distance travelled of the proposed approach can be evaluated by calculating the root mean squared error (RMSE) as the square root of the residuals with Equation (6):

(6) $$RMSE=\sqrt{\frac{\sum_{i=1}^{n}{\left(PS_{i}-SA_{i}\right)}^{2}}{n}}$$

where *n* represents the walking experiments conducted by each user along the given path. *PS_i_* denotes the final location predicted by the proposed algorithm, and *SA_i_* denotes the actual final location in the ith experiment.

**Figure 9:**
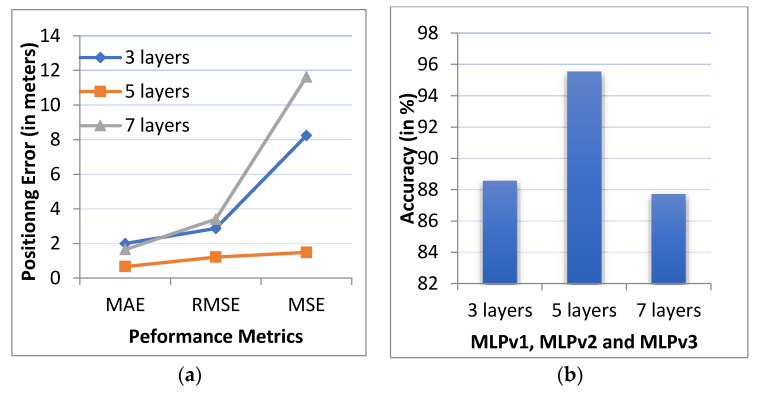
Comparison of: (**a**) the positioning error (MAE, MSE and RMSE) and (**b**) the accuracy for the number of the layers of the model.

We implemented three batch normalized versions of the MLP algorithm MLPv1, MLPv2, and MLPv3 with 3, 5, and 7 layers. All three versions were implemented in order to evaluate the performance of the best fit model, and to investigate the effects of a different number of hidden layers. The performance metrics, including MAE, MSE and RMSE, are shown in Figure 9a. The prediction accuracy of the model was evaluated by calculating the ratio of the number of correct prediction occurrences to the total number of predictions. The prediction accuracy is shown in Figure 9b.

The MAE positioning error for MLPv1 with three layers is 1.99 m, with a prediction accuracy of 88.57%. When the number of layers is increased to seven, the positioning error is reduced to 1.64 m, with a prediction accuracy of 87.71%. The performance of the MLPv2 with five hidden layers has a positioning error of 0.66 m, with a prediction accuracy of 95.54%. The results show that the average positioning error and prediction accuracy of the five layered network is better than the other two networks. As such, we use the network with five hidden layers in the rest of this work.

**Figure 10:**
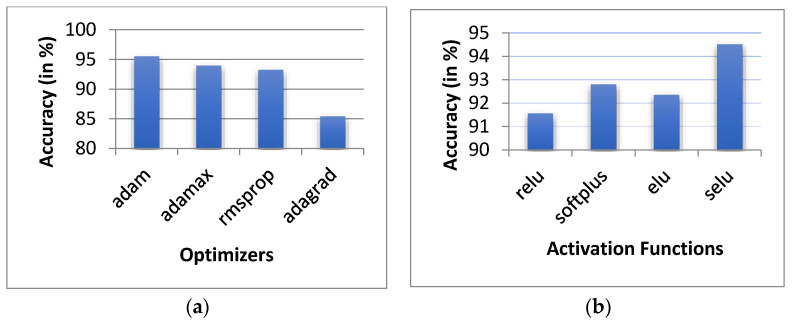
Prediction error with: (**a**) different optimizers and (**b**) different activation functions.

A different permutation of the hyperparameter was further implemented with a 5-layered network. As the input data is continuous and differentiating, we tested the model with non-linear (tanh, selu, relu and softmax and elu) activation functions. In the tuning process, we applied optimizers, including adam, adamax, rmsprop and adagrad. Table 2 shows the positioning errors for the variously-implemented optimizers and activation functions. For each optimizer, an appropriate learning rate was proposed. As shown in Table 1 and Figure 10a, the implementation of the adam optimizer provides the best performance, with an average of 0.71 m MAE error and 95.5% prediction accuracy. As such, we continued to keep the adaptive moment estimation (adam) with β1 = 0.9, for β2 = 0.999, and ϵ = (10 × exp (−8)).

The positioning accuracies using activation functions such as relu, softplus, elu and selu are shown in Table 2. All of the activation functions were performed with adam optimizer. It is evident from the results that the selu activation function outperforms the other activation functions, with a positioning error of 0.65 m and a 94.51% prediction accuracy. Table 2 shows that the minimum positioning error is found with the selu activation function, with 0.65 m as the MAE, 1.67 m as MSE and 1.29 m as RMSE.

From Figure 10b and Table 2, we can conclude that the selu activation function outperforms the relu, softplus and elu activation functions.

**Figure 11:**
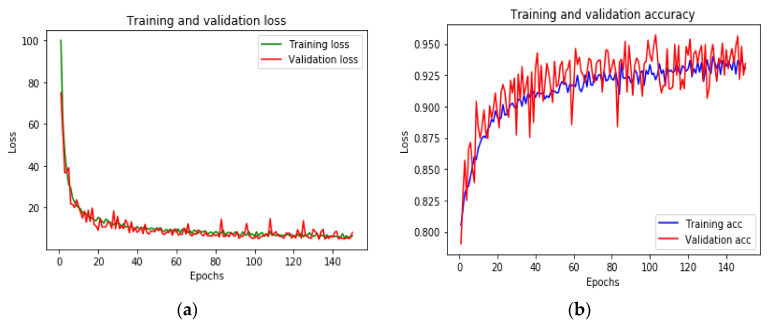
Training and validation (**a**) loss and (**b**) accuracy for the deep MLP model.

The loss function declining curve with different epochs on the training and validation dataset is plotted in Figure 11a,b. It clearly shows that the curve becomes stable after 60 epochs, but we continued the observation to the 140th epoch.

**Figure 12:**
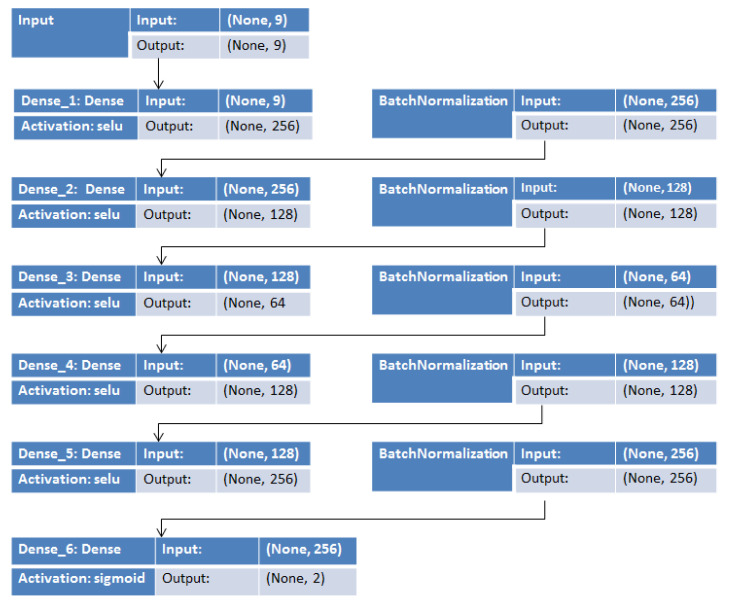
The best-suited regression-based deep neural network MLP model.

**Figure 13:**
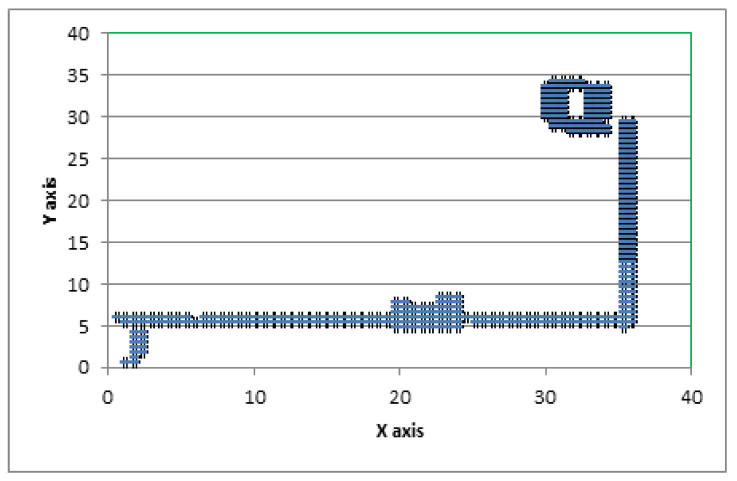
Actual (*x*, *y*) position based on the dataset.

**Figure 14:**
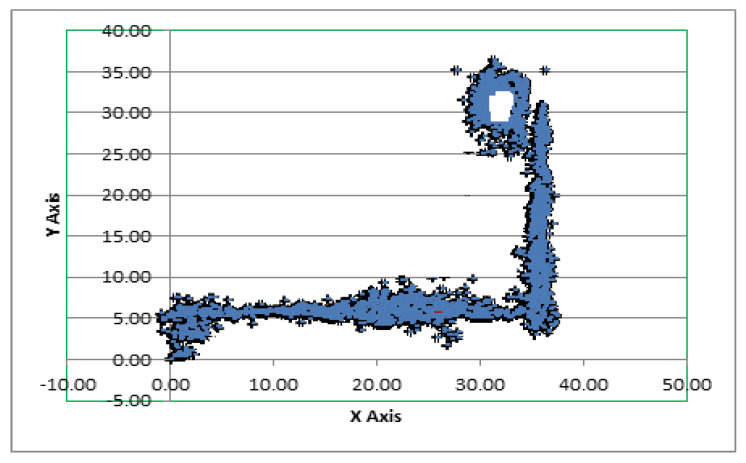
Predicted (*x*′, *y*′) position based on the deep MLP model.

Figure 12 represents the best-suited regression-based deep neural network model used for the predictions. Figure 13 represents traces of the actual position (*x*, *y*) from the IPIN2016 dataset for user 1. Figure 14 shows the calculated predicted positions (*x*′, *y*′) using the best-suited regression MLP model.

The results demonstrate that the proposed model achieves a considerable prediction accuracy of 94.51%, with a 0.65 m positioning error. The highest positioning error is not higher than 0.89 m. The training time for the given model is approximately 16 s, and the prediction time for a given sample, once trained, is 5 ms. Our previous work based on an improved positioning algorithm [43], when applied on the dataset, provides an almost equal prediction accuracy of 95%. However, the positioning error was evaluated as 1.5 to 2 m. The method proposed in [10] needs an absolute position from additional devices, such as a those that are capable of generating beacon signals. The variation of the error is too high compared to the proposed model. Moreover, the computation time is almost doubled, to 10 to 12 ms.

## 6. Conclusions

This paper proposed a novel approach to achieve the positioning of a moving VI person as part of an indoor navigation system. The approach is based on feeding the data from the inertial sensors of a typical smartphone to a trained MLP that will map them into the 2D local coordinates of the microcell corresponding to the position of the person holding the phone. The proposed approach was tested with the data from a publicly available multivariant dataset, IPIN2016. The dataset contains data from movements that resemble the walking of a VI person. The performed experiments show that the proposed approach is capable of achieving a positioning accuracy that is close to the step size of a typical user: around 0.65 m. We performed our experiments on a grid size of 0.6 by 0.6 m. Our future work will investigate the impact of different grid sizes on the positioning error and prediction accuracy. We also intend to test our approach using a much larger, temporal dataset to observe the impact of magnetic intensity variations on the prediction error. It is noted that the proposed approach requires Internet connectivity, as it relies on the receipt of the position estimates from the trained model residing in the cloud. In our future works, we intend to explore whether this shortcoming can be addressed by using pre-trained models in a smartphone app. We also aim to complement our proposed positioning approach with other navigation components in order to facilitate easy indoor movements by a VI person.

## Figures and Tables

**Table 1:** The experimental model with the hyperparameter values.

Parameter	Hyperparameter Values in Proposed Deep MLP
Software	Python, Keras, TensorFlow
Training data	29,436
Validation data	7359
Epochs	60 to 140
Batch size	20, 40, 60, 80
Layers with Hidden neurons (with batch normalization)	3 layers—128, 64 and 128 neurons 5 layers—256, 128, 64 and 128 and 256 neurons 7 layers—512, 256, 64, 128, 256 neurons
Drop out rate	0.2 to 0.8
Activation	Selu, elu, softplus, relu
Optimizer	Adam, adamax, rmsprop, adagrad
Loss function	MAE, MSE, RMSE

**Table 2:** Positioning error (in meters (m)) with different optimizers and activation functions.

**Optimizer**	**MAE (m)**	**RMSE (m)**	**MSE (m)**
**Adam**	0.71	1.30	1.70
**Adamax**	0.84	1.35	1.81
**Rmsprop**	1.04	1.84	3.39
**Adagrad**	5.59	3.25	3.62
**Activation**	**MAE (m)**	**RMSE (m)**	**MSE (m)**
**relu**	1.24	2.61	6.84
**softplus**	1.35	2.45	6.01
**elu**	0.92	1.85	3.45
**selu**	0.65	1.29	1.67
